# Different Time Scale Distribution of Negative Air Ions Concentrations in Mount Wuyi National Park

**DOI:** 10.3390/ijerph18095037

**Published:** 2021-05-10

**Authors:** Changshun Li, Ziyang Xie, Bo Chen, Kaijin Kuang, Daowei Xu, Jinfu Liu, Zhongsheng He

**Affiliations:** 1 Fujian Meteorological Service Center, Fuzhou 350001, China; Lchangshun@163.com; 2 Institute of Meteorological Big Data-Digital Fujian, Minnan Normal University, Zhangzhou 363000, China; 3 College of Forestry, Fujian Agriculture and Forestry University, Fuzhou 350002, China; nmgxzy94@126.com (Z.X.); cb137751120@163.com (B.C.); kkj1205@163.com (K.K.); xudaowei2004446@126.com (D.X.); fjljf@126.com (J.L.); 4 Key Laboratory of Fujian Universities for Ecology and Resource Statistics, Fuzhou 350002, China

**Keywords:** negative air ions (NAIs), time series, ARIMA model, health

## Abstract

The concentration of negative air ions (NAIs) is an important indicator of air quality. Here, we analyzed the distribution patterns of negative air ion (NAI) concentrations at different time scales using statistical methods; then described the contribution of meteorological factors of the different season to the concentration of NAIs using correlation analysis and regression analysis; and finally made the outlook for the trends of NAI concentrations in the prospective using the auto regressive integrated moving average (ARIMA) models. The dataset of NAI concentrations and meteorological factors measured at the fixed stations in the Mountain Wuyi National Park were obtained from the Fujian Provincial Meteorological Bureau. The study showed that NAI concentrations were correlated with relative humidity spanning all seasons. Water was an important factor affecting the distribution of NAI concentrations in different time series. Compared with other ARIMA models, the outlook value of the ARIMA (*0,* *1*, *1*) model was closer to the original data and the errors were smaller. This article provided a unique perspective on the study of the distribution of negative air oxygen ions over time series.

## 1. Introduction

Negative air ions (NAIs) is a generic term for negatively charged gas molecules and ions [1]. Negative air ions are also known as negative air (oxygen) ions, since they form negative oxygen ions based on their ability to acquire electrons, most of which are acquired by oxygen. In this study, NAIs referred mainly to oxygen-based negative ions. A study showed that superoxide ions were involved in the biological effects of NAIs [2]. NAIs also have many beneficial effects on human health, including physically and psychologically. On the physical side, NAIs have a beneficial effect on the cardiovascular [3,4] and respiratory systems [5]. On the psychological side, NAIs can improve sleep quality [6], improve mood states [7] and treat chronic depression [8]. NAIs were mainly generated by the following pathways: cosmic ray [9], radiation emitted by the radon element of mineral [10], solar ultraviolet radiation [11], thunder and lightning [12], the shearing forces of water (the Lenard effect) [3] and plants [13]. Therefore, factors such as vegetation cover, flowing water bodies, and air humidity could be considered an important influence on the anion content [14]. Moreover, forest health has emerged as a popular health and wellness program in China today due to a large amount of NAIs produced by forests.

**Figure 1:**
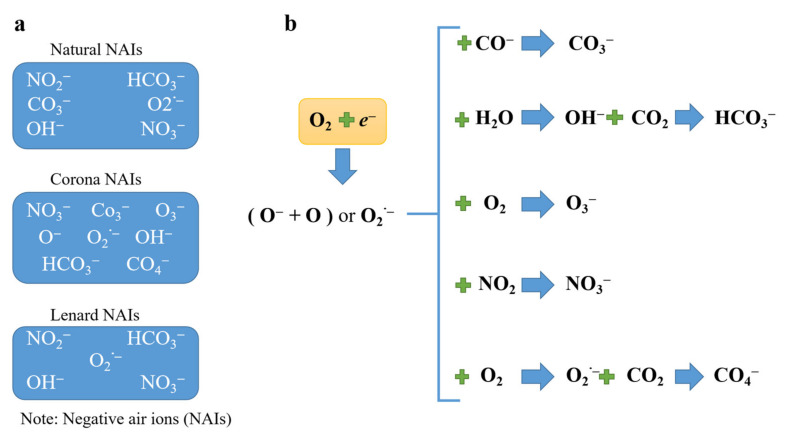
The types of NAIs generated through different ways through the oxygen − based (**a**) NAI compositions. (**b**) The evolution formula of NAIs. The blue arrows indicate the NAI transformation processes.

We referred to the formulas and pictures generated by NAI in the article by Jiang Shuye [14], and list the ways, types and formulas of NAIs generation (Figure 1). The types of NAIs produced in different ways and their main compositions are natural NAIs [3,15,16,17], corona NAIs (generated by the corona discharge ionization) [18,19,20,21,22] and Lenard NAIs (generated by the shearing force of water) [23,24,25], as shown in Figure 1a.

Figure 1b shows the evolution of a NAI to form another NAI. As a result, other NAIs are generated such as CO_3_^−^, OH^−^, HCO_3_^−^, O_3_^−^, O_2_^−^, CO_4_^−^ and NO_3_^−^ [16,17,23,26,27,28]. In addition, the composition of NAIs is dynamic in the air, which depends on the electron affinity and potential [29]. The equation in Figure 1 shows that the negative ions of oxygen react with water, so we introduce meteorological elements in the following part of this paper with a view to analyzing the relationship between meteorological factors and NAIs.

Mount Wuyi National Park is densely forested and characterized by high vegetation cover. A few studies have been conducted on studying the systematic production of negative oxygen ions in Mount Wuyi National Park. Studies on NAIs have mainly focused on the coupling relationship between NAIs and correlated factors [30] and the spatiotemporal distribution pattern of NAIs [31], and NAIs also used in medical and chemical applications [32,33].

Here, we launched a study on the temporal distribution pattern of NAI concentration in Mount Wuyi National Park and based on the auto regressive integrated moving average model (ARIMA) time series model, we made an outlook for the value of NAI concentrations in this area, with the week as the time unit. Specifically, we used the average NAI concentrations from week 1 to week 55 (1 October 2018 to 20 October 2019) as a training sample to make the outlook for the average NAI concentrations from week 56 to week 68 (21 October 2019 to 19 January 2020). Our purpose is to cover the gap in NAIs research in Mount Wuyi National Park and provide theoretical guidance for describing the NAI concentration, providing an outlook for the changing trend of NAI concentration.

## 2. Methods

### 2.1. Study Site Selection

The study was conducted in the Mount Wuyi National Park of Fujian Province in China. We set the Atmospheric Negative Air Ions Monitoring Station (117°24′12″, 27°32′36″) in the Mount Wuyi National Park, which can monitor the real-time concentration of NAIs for 24 h and represent the concentration of NAIs in the park. The data collection period ranged from 1 October 2018 00:00 to 20 February 2020 20:00 to calculate the NAI concentrations on different time scales. Besides, the meteorological data were collected from the adjacent site less than 100 m away to investigate the influence of meteorological factors on NAIs.

### 2.2. Instrumentation

**Figure 2:**
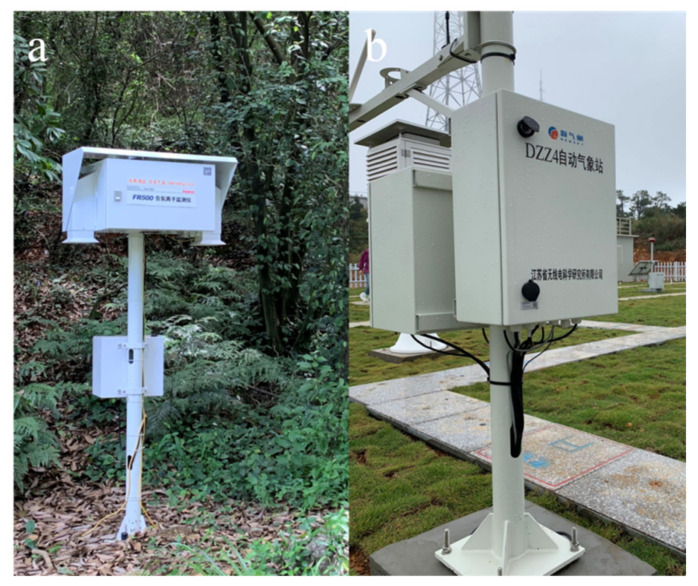
Meteorological observation system, including; (**a**) FR500 negative oxygen ion monitor, and (**b**) DZZ4 automatic meteorological station.

The average NAI concentrations were measured by the FR500 negative oxygen ion monitor (Huatron Corporation, Beijing, China) under the NAIs list by the China Meteorological Administration, as shown in Figure 2a. This instrument is usually calibrated in real-time and has a high measurement accuracy of ≤5%. The measuring range was: 0–50,000 ion/cm^3^; ion mobility: ≥0.4 cm^2^/ (V.s). The instrument meets the requirements of the functional specifications of the China Meteorological Administration.

We used the DZZ4 automatic meteorological station, as shown in Figure 2b (Jiangsu Radio Scientific Institute CO., LTD, Nanjing, China), to measure the following meteorological factors: air temperature, atmospheric pressure, relative humidity, precipitation, wind speed and visibility. The parameters were as follows: precipitation in 1 h (PRE, mm); average temperatures (TEM, °C); average atmospheric pressure (PRS, hpa); average relative humidity (RHU, %); average wind speed of 10 min to be averaged in 1 h (WIN, m/s) and visibility (VIS, m).

### 2.3. Data Selection

Here, we developed a statistical method to calculate the average NAIs on different time scales based on the raw hourly data. For the raw hourly data, there were 12,190 values, with 10,681 valid values and 1509 missing values. We used the raw valid values for statistical analysis.

(1) Average hourly value. We calculated the average daily hourly value (0:00–23:00) by averaging the valid values (excluding missing values) of all hourly data from 1 October 2018 0:00 to 20 February 2020 22:00. Specifically, we calculated the value of NAIs per hour in an average of 1 day during this period. We took all the data holidays for an hour in this period (1 October 2018 0:00 to 20 February 2020 22:00) and divided by the total number.

(2) Daily value (00:00–23:00). We calculated the mean of the daily data using valid hourly data. 

(3) Weekly value. The average of the hourly value during the week was used as the weekly value. The study delineated a range of 1 to 68 weeks based on specific dates, as shown in Table S1.

(4) Monthly value. The average of the daily value during the month was used as the monthly value.

(5) Frequency of NAI concentration intervals. The study used a frequency histogram to analyze the frequency distribution of different NAI concentration intervals.

Since meteorological factors and seasonal factors could influence the NAI concentrations, we used the meteorological data to investigate the effect of meteorological parameters on NAIs. We used the raw meteorological data: hourly valid data for the period from 1 December 2018 0:00 to 30 November 2019 23:00 and then divided this time period into 4 seasons (winter season from 1 December 2018 to 28 February 2019, spring season from 1 March 2019 to 31 May 2019, summer season from 1 June 2019 to 31 August 2019 and fall season from 1 September 2019 to 30 November 2019). Pearson correlation analysis and multiple linear regression analysis of raw valid hourly data of the meteorological data and NAI concentrations data in four seasonal periods was carried out. We here aim to explore which meteorological factors contributed to NAI concentrations in different seasons. Pearson correlation analysis and multiple linear regression analysis were carried out using SPSS 14 software (IBM SPSS, Armonk, NY, USA).

### 2.4. Model Selection

The concept of the auto regressive integrated moving average (ARIMA) model was proposed by Box and Jenkins in the 1970s [34,35]. For the ARIMA (*p*, *d*, *q*), AR shows auto-regression, MA presents moving average, *p* presents the number of terms of auto-regression of the model, *q* presents the moving average term of the model and *d* shows difference times [36].

(1) $$Y_{t}=c+\emptyset_{1}\;y_{t-1}+\ldots +\emptyset_{p}\;y_{t-p}+\theta_{1}\;y_{t-1}+\ldots +\theta_{q}\;\varepsilon_{t-q}+\varepsilon_{t}$$

In the formula, *Y*_t_ presents differentiated sequence, $\varepsilon_{t}$ is the noise sequence, ∅ is the fitting parameter of the AR model, and θ is the fitting parameter of the MA model.

**Figure 3:**
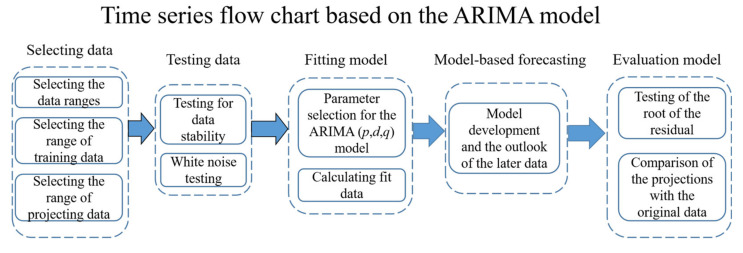
ARIMA model flow chart.

The study is based on the R software “forecast” and “tseries” packages to construct the ARIMA time series model (Figure 3). The building process of the ARIMA model includes the following parts.

(1) Data selection. The study selected weekly data as the unit based on the hourly data from 1 October 2018 0:00–19 January 2020 23:00. The weekly data were divided into 68 weeks. The 1 to 55 weeks were training data to predict the NAIs from 56 to 68 weeks, and the actual value was used as one of the criteria to evaluate the accuracy of the outlook. The data for week 28 was null, and we took the predicted value of the ARIMA model in the short-range to fill the missing values using the data from week 1 to week 27 by comparing the measurement information of different ARIMA models.

(2) Data testing. We conducted the smoothness and the white noise test on the selected data. If the series did not pass the smoothness test, we made it different to obtain a smooth time series; then we conducted the white noise test on the data. The white noise series is a sequence of independent random identically distributed and zero-mean variables [37]. If the smooth data series was a white noise series, the series variation had no regularity, so the trend of the series could not be found, and the ARIMA model’s construction is meaningless [38]. If the result was smooth non-white noise data, then were able to fit the model. We used R package “stats” to perform white noise detection on the time series and “tseries” to perform smoothness tests.

(3) Model Fitting. For determining the parameters (*p*, *d*, *q*) in ARIMA, if the data were differenced in the previous step, then the parameter *d* is the number of times the difference was made. The model with the most appropriate *p* and *q* values was then selected by comparing the relevant values’ size (Table 1). The AIC criterion was proposed by the Japanese statistician Hiroshi Akaike in 1974 [39]; it was based on the concept of entropy. It provided a measure for weighing the estimated model’s complexity and the goodness of fit of the data. The best model is selected by building a model with different parameter values: (*p*, *q*) of ARIMA. The smaller the AIC value was, the better the model would be. The following was the AIC guideline definition formula.

(2) $$AIC\left(n,m\right)=\ln{\hat{\sigma }}_{\alpha }^{2}+2\left(n+m+1\right)/N$$

If *AIC*(*p*, *q*) =$\underset{0\leq n,m\leq 1}{\min}AIC\left(n,m\right)$, then the ARMA model order is determined to be (*p*, *q*) and $\sigma_{\propto }^{2}$ was calculated by maximum likelihood estimate (MLE). The purpose of MLE was to maximize the probability of the selected sample appearing in the selected population. The AIC criterion has the advantage of reducing the influence of subjective factors on the model’s accuracy and allows the determination of *p* and *q*. The AIC criterion performs a series maximum likelihood estimate of the time series and requires the data to satisfy a normal distribution.

We filtered the most appropriate *p* and *q* values by the AIC criterion, and further filtered the model with reference to the relevant statistics such as the square root RMSE of the mean squared error and the MAPE of the mean absolute percentage error. We used the R package “base”to make the difference of the time series and “forecast” to fit the model and evaluate accuracy.

(4) The outlook of the later values of NAI concentrations and evaluation of the model. For the outlook of time series, we used the ARIMA model. Specifically, the ARIMA model is trained on the original data to predict the time series’ future trend. After the outlook of the later values, we evaluated the model in 2 steps: white noise detection of residuals and normal distribution detection of residuals. (a) The residuals’ white noise test was based on the Ljung–Box method to determine if the *p*-value was higher than 0.05 and the residuals were white (we would prefer the residuals to be white noise) and the result passed the white noise test. (b) For the residuals’ normal distribution test, we used the R package “ggpubr” and “ggplot2” to plot a normal QQ plot of the residuals and visually determine if most of the drop points were on or near the line. If the residuals pass (a) and (b), it means that ARIMA can fit the data successfully.

## 3. Results

### 3.1. The Distribution Characteristics of NAIs at Different Time Scales

**Figure 4:**
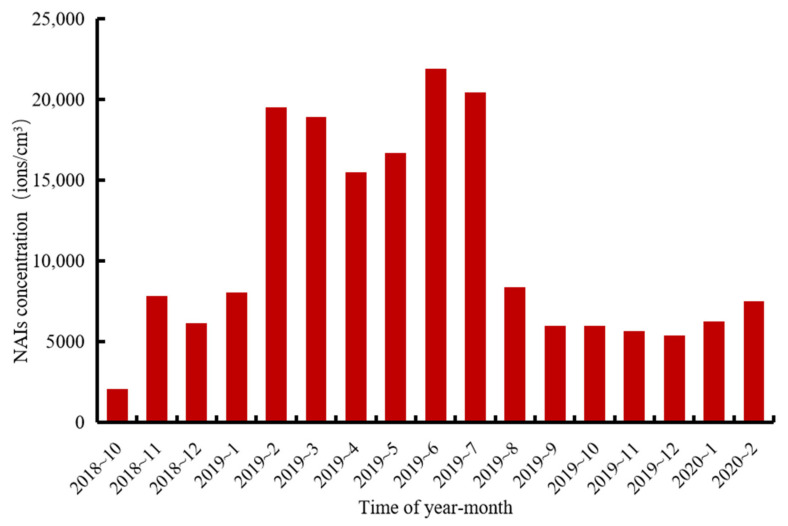
Monthly average NAI concentrations from October 2018 to February 2020.

By analyzing the monthly data of NAI concentrations from October 2018 to January 2020 (as shown in Figure 4), we could see that NAI concentrations from February to July 2019 were much higher than in other time periods, while NAI concentrations were low in October 2018 to January 2019 and August 2019 to February 2020. The above two figures showed clear seasonality in the monthly distribution of NAIs concentrations, with higher concentrations in spring and summer and lower concentrations in autumn and winter. We also found that the lower NAI concentrations occurred in the summer months of August.

The NAI concentration value of August 2019 was much lower than July 2019. We further verified this phenomenon using correlation analysis between NAIs and meteorological factors in Table 1 based on raw hourly data. Specifically, we divided 1 December 2018 at 0:00 to 30 November 2019 into four parts according to the seasons. From 1 December 2018 to 28 February 2019 was defined as winter, from 1 March 2019 to 31 May 2019 was defined as spring, from 1 June 2019 to 31 August 2019 was defined as summer and from 1 September 2019 to 30 November 2019 was defined as autumn. We matched hourly data for meteorological factors with hourly data for NAIs on a time series. For missing values of NAIs, we censored the meteorological data for the same time and matched them.

The results showed that different meteorological factors influenced NAIs in different seasons, with relative humidity being significantly (positively) correlated (*p* < 0.01) with NAI concentrations in all seasons. The (positive) correlation (*p* < 0.05) between precipitation and NAI concentrations occurred only in spring. The fact that rainfall had less impact on NAI concentrations was due to the drought suffered in summer and autumn 2019, as shown in Table 1. Remarkably, the highest the value of Pearson correlation for meteorological factors was 0.306 from RHU in winter, so we used a multiple linear regression model to further explore the contribution of meteorological factors to NAIs.

The study further analyzed the contribution of the meteorological factors with *p* < 0.01 in Table 1 to the NAIs using the multiple linear regression model as shown in Table 2. The meteorological factors in winter contributed the most to NAI concentrations, and the value of R^2^ was 0.224. Meteorological factors in other seasons contributed less to NAI concentrations.

**Figure 5:**
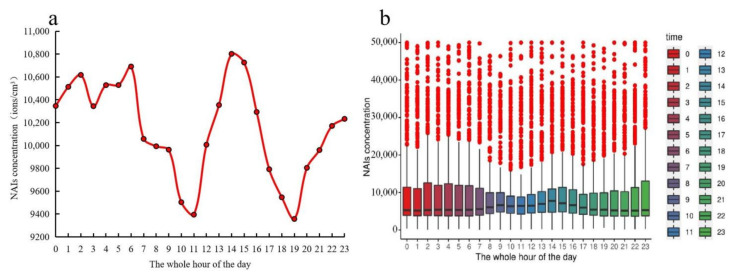
Daily distribution of hour-by-hour value of NAI concentrations. (**a**) Average hourly value of NAI concentration distribution a day. (**b**) Boxplot of NAI concentrations of raw hourly data. The value of the boxplot includes outliers, maximum, third quartile, median, first quartile and minimum.

The average hourly value of NAI concentration distribution a day showed an approximate increasing trend from 19:00–6:00 and was much higher in 6:00 and 14:00–15:00. Overall, NAI concentrations were higher at midday and in the afternoon and were lower in 10:00–11:00 in the morning and 19:00 (Figure 5a). Due to the excessive amount of data, the data overlaps, so we calculated the mathematical statistics for the raw hourly data based on the boxplot (Figure 5b). The value of the boxplot includes the maximum, third quartile, medium, first quartile, minimum and outliers. The maximum NAI concentration in the figure is 50,000, which is because the range of the negative oxygen ion monitoring instrument that can be monitored is 0–50,000. We can see that the value of the median, maximum, third quartile and first quartile of the hour of the day was roughly similar to the trend in Figure 5a.

**Figure 6:**
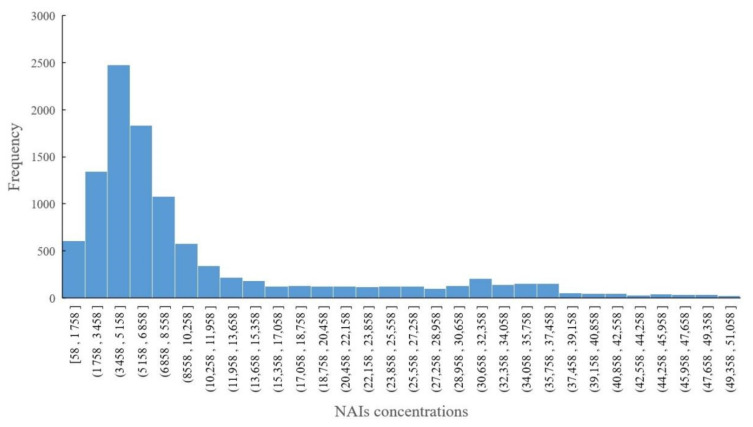
Frequency histogram of hourly data of NAI concentrations.

We calculated the frequency of NAI concentration intervals based on hourly data from 1 November 2018 00:00 to 20 February 2020 20:00, as shown in Figure 6. The NAI concentration range of its frequency over 500 includes [58, 1758], (1758, 3458], (3458, 5158], (5158, 6858], (6858, 8558] and (8558, 10,258]; these interval segments are connected to each other, and connecting them to each other forms a new interval, which is (58, 10,258). Among [58, 10258], the interval [3458, 5158] has the highest frequency, reaching 2477. These results show that as the concentration of NAIs increases, the frequency also becomes very low, and the frequency of outliers in NAI concentrations is also relatively small. 

### 3.2. Filling in Missing Values of NAI Concentrations by Developing ARIMA Time Series Models

We used the average weekly value as weekly values and combined them into 68 weeks of data. The first 55 weeks were the training data, and the NAIs were predicted for 56–68 weeks.

The data for week 28 were null, and we took the ARIMA model in the short-range to fill the missing values from the R package “forecast”. We used the predicted value output by the ARIMA model to fill in missed values. First, we performed the stationarity test and white noise test on the data of 1–27 weeks and fitted the ARIMA (*p*, *d*, *q*) model. The smoothness test result was *p*-value = 0.4322 > 0.05, so we needed to perform one differentiation in the time series. After one differentiation, we secondly performed the smoothness test and the result was *p*-value = 0.05, which passed the smoothness test, but it did not pass the white noise test because *p*-value = 0.08486 > 0.05. The new series passed the smoothness test with *p*-value = 0.01203 < 0.05 and the white noise test with *p* = 0.004859 < 0.05. Finally, we defined the *d* value of ARIMA (*p*, *d*, *q*), which was 2.

We fitted different ARIMA models to compare their accuracy measures, as shown in Table 3. For parameter *p* and *q*, we simulated the *p*, *q* values in different models and chose the most appropriate model by comparing the Akaike information criterion (AIC), mean error (ME), root mean squared error (RMSE), mean absolute error (MAE), maximum permissible error (MPE), mean absolute percentage error (MAPE, %), the mean absolute scaled error (MASE) and ACF1 (auto correlation of errors at lag 1). We found that the RMSE and AIC was smallest when (*p*, *d*, *q*) was (*0*, *2*, *2*) and used 2,6238.82 to be the predicted value of this model to fill the missed value.

After completing the model, we tested the fit model consisting of a white noise test on the residual test series. The “white noise” was different from the white noise before modeling, and we hoped that the residuals of the model were completely random series, i.e., the white noise series. The residual white noise of ARIMA (*0, 2, 2*) model was tested by the Ljung–Box test; the *p*-value was 0.803, which indicated that the residuals were not autocorrelated, and the residual was a sequence of the white noise.

**Figure 7:**
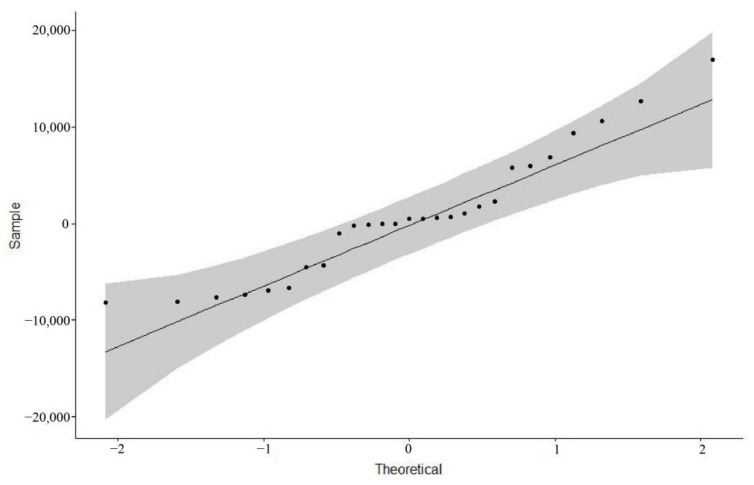
The normal distribution QQ plot of the residuals of the ARIMA (*0*, *2*, *2*) model.

Then, we tested the residuals of the ARIMA (*0, 2, 2*) model for normality by plotting a QQ plot of the normality test, by drawing a 45-degree line relative to the x and y axes and visually observing whether the points representing the residuals fell on or near the 45-degree line, and the grey area in the graph represents the 95% confidence interval of the normal distribution (Figure 7). All values were inside the grey area. Our result indicated that the residuals passed the normality test and showed that the established ARIMA (*0, 2, 2*) model was reasonable.

### 3.3. Making the Outlook of NAIs Concentrations by Developing ARIMA Time Series Models

After filling in the missing data from week 28 using ARIMA (*0*, *2*, *2*), we started to make the outlook the data from weeks 56–68 based on the ARIMA model for data from weeks 1–55. According to the smoothness test for the raw data, its *p*-value was 0.6033, which was greater than 0.05, then it failed to do the smoothness test. We needed to differentiate the data to make it stable.

Next, we differenced the data once, then the *p*-value was less than 0.05, which passed the smoothness test, and the differenced time series was a smooth time series. The differential data were tested for white noise (*p*-value = 0.002258) and passed the white noise test, so the data were smooth non-white noise data and could be fitted to the ARIMA model.

Here, we selected the ARIMA (*p*, *d*, *q*) model parameters, where *d* = 1 since the previous data were differenced once. For parameter *p* and *q*, we simulated the *p*, *q* values in different models and chose the most appropriate model by comparing the Akaike information criterion (AIC), mean error (ME), root mean squared error (RMSE), mean absolute error (MAE), maximum permissible error (MPE), mean absolute percentage error (MAPE, %), the mean absolute scaled error (MASE) and ACF1 (auto correlation of errors at lag 1), as shown in Table 4. We found that the RMSE and AIC was smallest when (*p*, *d*, *q*) was (*1*, *1*, *1*), (*0*, *1*, *1*), (*0*, *1*, *2*), (*2, 1, 0*) and *(2*, *1, 1*). Combining the other metrics, we finally chose (*0*, *1*, *1*) as the (*p*, *d*, *q*) value of the model.

**Figure 8:**
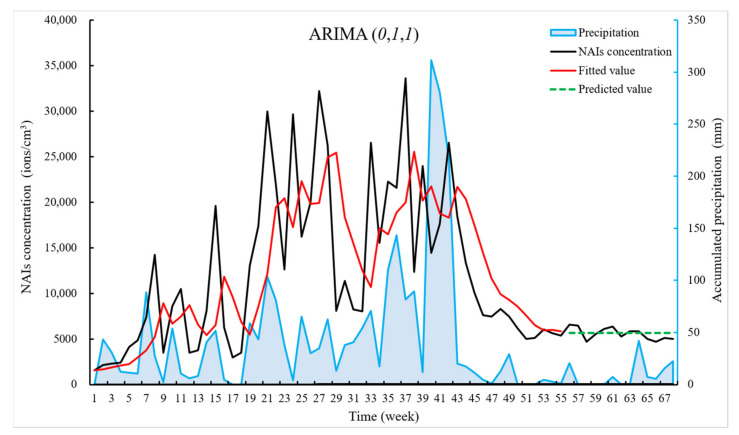
Time series of weekly data of NAI concentration. Week 1 to 55 was used as a time series of training data. We can see that the time series was highly fluctuating and uneven, and combined with topic 3.2, where the data did not pass the smoothness test, we made 1 difference to the series to make the series smooth, which was the red line. In the ARIMA (*0, 1, 1*) model time series plot, the black line represented the raw data of NAI concentrations, and the green line represented the predicted values of NAI concentrations, which were closer to the raw values. The blue line represents weekly precipitation (accumulate hourly precipitation into weekly precipitation).

We predicted NAI concentrations for 56–68 weeks and plotted them in a time series plot based on the ARIMA (*0, 1, 1*) model, as shown in Figure 8. ARIMA (*0, 1, 1*), compared to the other ARIMA series model, had fitted values for 1–55 weeks that were relatively rightward skewed due to the one difference, which had less impact on the results. The ARIMA (*0, 1, 1*) model had relatively low values of AIC, RMSE, etc., and could largely predict trends in NAIs well over the next 13 weeks. 

Besides, the study found that the weekly precipitation and weekly NAI concentration have similar trends in the time series. Both precipitation and NAIs show a low and gentle trend in the later periods, as shown in Figure 8. There may be some connection between NAIs and the weekly data of accumulated precipitation. The meteorological factors were linked to the NAIs in some way, so we next tested the weekly data on meteorological factors and NAIs for correlation as shown in Table 5.

As seen in Table 5, the study showed a significant correlation between the concentrations of NAIs and cumulative precipitation, mean atmospheric pressure, and mean relative humidity.

To further explore the contribution of meteorological factors to weekly NAI concentrations, we performed a multiple linear regression analysis using the meteorological factors with the highest correlations in Table 6 (PRE_W, PRS_W, RHU_W) and NAI concentrations. We can see that the value of R^2^ is 0.349. This means that the meteorological factors (PRE_W, PRS_W and RHU_W) contribute 34.9% to NAIs.

After completing the model, we tested the fit model consisting of a white noise test on the residual test series. The “white noise” was different from the white noise before modeling, and we hoped that the residuals of the model are completely random series, i.e., the white noise series. The residual white noise of the ARIMA (*0, 1, 1*) model was tested by the Ljung–Box test; the *p*-value was 0.9571, which indicated that the residuals were not autocorrelated, and the residual was a sequence of the white noise.

**Figure 9:**
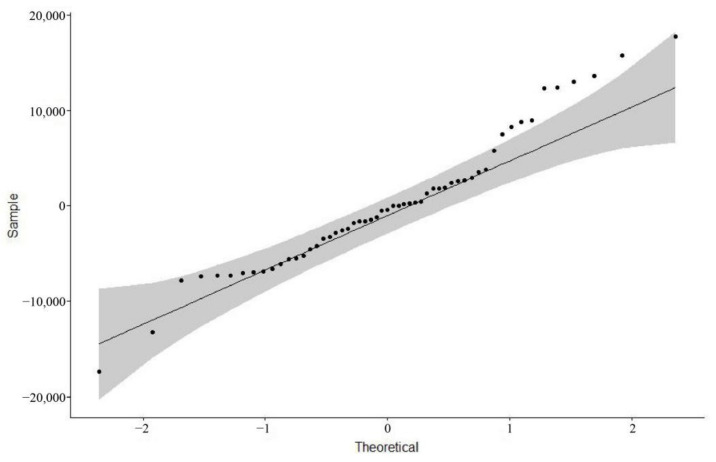
The normal distribution QQ plot of the residuals of the ARIMA (*0*, *1*, *1*) model.

Then, we tested the residuals of the ARIMA (*0, 1, 1*) model for normality by plotting a QQ plot of the normality test, by drawing a 45-degree line relative to the x and y axes and visually observing whether the points representing the residuals fell on or near the 45-degree line, and the grey area in the graph represents the 95% confidence interval of the normal distribution (Figure 9). Although a small number of values were outside the gray area, most values were around the 45% line and inside the grey area. Our result indicated that the residuals largely passed the normality test. Overall, we used the ARIMA (*0*, *2*, *2*) model to fill in the week 28 data based on the weeks 1–27 data. Then, the ARIMA (*0*, *1*, *1*) model was used to predict the week 56–68 data based on the week 1–55 data. Our results showed that the established ARIMA (*0*, *2*, *2*) and ARIMA (*0, 1, 1*) model was reasonable.

## 4. Discussion

### 4.1. Time Series Analysis Based on Available Data

We found that NAI concentrations have a distinct diurnal variation profile. Specifically, NAI concentrations were highest at 0:00–6:00 and 14:00–15:00 daily and lowered at 10:00–11:00 and 18:00–19:00. The distribution of other meteorological factors may cause this during the day. 

The monthly variation in NAI concentrations showed clear variation characteristics, with lower concentrations of NAIs from October 2018 to January 2019 and August 2019 to February 2020, and higher concentrations from February 2019 to July 2019.

The study found that the NAI concentrations and the cumulative precipitation have similar trends in the weekly time series, which shows certain links (Figure 8). The study conducted correlation analysis and multiple linear regression analysis between NAIs and meteorological factors on both hourly and weekly data and found significant correlations between NAIs and relative air humidity on hourly time scales, and between NAIs and cumulative precipitation, relative humidity and barometric pressure on weekly data scales. Water is an important factor in the concentration of NAIs. In addition, it can be seen from the formula of oxygen-based NAIs that water can produce NAIs by Lenard force, and oxygen-based NAIs can react with water (Figure 1). This was due to the severe lack of precipitation in the Wuyi mountains from August 2019, resulting in lower air humidity and lower biological activity, which has reduced the amount of water and plant-produced NAIs.

The study also found that the trend of NAI concentrations in some places in the figure slightly lags the trend of precipitation. The reasons for the lag are: (1) According to the maintenance personnel, water easily entered the negative ion monitor and damaged it when in the high precipitation, and then the instrument went to be repaired, which makes the observation of negative oxygen ions missing during this period of time. (2) After rainy days, the water of precipitation evaporated on a sunny day, which also increased relative humidity and the NAI concentrations of the air.

### 4.2. Research on the ARIMA Model Methodology

The ARIMA (*0*, *1*, *1*) model, which predicted a gradual increase in air anion concentration over the next 13 weeks in weekly time units, performed well in the long-range prediction, and the predicted values were close to the original values in terms of magnitude and trend. However, in terms of the order of magnitude and trend, the predicted values are becoming stable later as time increased, which may be related to the fact that the data undergo one difference and the sparse amount of data. Besides, there was a clear deviation between the predicted and actual values after week 68 in terms of values and trends, which may be related to other influencing factors and the application of the model to short- and medium-range outlooks. Further research is needed to model the long-range prediction of NAI concentrations and analyze the related influence mechanisms. Long-range trend predictions will be challenging, given the climate changing and human activities which can impact multiple relevant environmental factors related to NAIs and no single statistical model that can fully and clearly assess such complex interactions. Meteorological factors also should be considered in forecasting models. According to this study, future predictions of NAI concentrations in the Wuyi Mountain can be combined with predictions of precipitation. Our research has attempted to decompose seasonal factors, but the fitting failed due to the limitation of the period of the raw data. Future studies can also consider external variables such as seasonal factors and weather factors in the prediction model. Due to the small amount of time series data in the study, it is unreasonable to clearly define the impact that seasonality will have on the distribution of NAIs. For seasonality, it is recommended that seasonality be determined from data measured over a period of 5–10 years or more.

## 5. Conclusions

This study described the distribution of NAI concentrations over time using mathematical statistics and investigated the correlation of meteorological factors on NAI concentrations. Water was a significant factor in the temporal distribution of NAI concentrations. In addition, we attempted to use ARIMA models to provide an outlook on later data of NAI concentrations. This article provided a unique perspective on the study of the distribution of negative air oxygen ions over time series.

## Figures and Tables

**Table 1:** Pearson correlation between NAI concentrations and meteorological factors based on raw hourly data.

Season	Correlation and Significance	PRE	TEM	PRS	RHU	WIN	VIS
Spring	Pearson correlation	−0.052 *	−0.034	0.136 **	−0.070 **	0.004	0.082 **
	Significance	0.031	0.155	0.000	0.004	0.876	0.001
	*n*	1699	1699	1699	1699	1699	1699
Summer	Pearson correlation	0.002	−0.117 **	−0.008	0.140 **	−0.096 **	−0.026
	Significance	0.931	0.000	0.735	0.000	0.000	0.303
	*n*	1608	1608	1608	1608	1608	1608
Autumn	Pearson correlation	0.023	0.020	−0.112 **	0.150 **	−0.099 **	0.030
	Significance	0.302	0.357	0.000	0.000	0.000	0.181
	*n*	2051	2051	2051	2051	2051	2051
Winter	Pearson correlation	−0.017	−0.116 **	−0.179 **	0.306 **	−0.061 **	0.127 **
	Significance	0.448	0.000	0.000	0.000	0.006	0.000
	*n*	2028	2028	2028	2028	2028	2028

Note: ** *p* < 0.01; * *p* < 0.05. The parameters were as follows: precipitation per hour (PRE, mm); average air temperatures (TEM, °C); average atmospheric pressure (PRS, hpa); average relative humidity (RHU, %); average wind speed of 10 min (WIN, m/s) and visibility (VIS, m).

**Table 2:** Multiple linear regression analysis of meteorological factors and NAIs in different seasons.

Season	R	R^2^	Durbin-Watson	Meteorological Factors
Spring	0.160	0.026	0.340	PRS; RHU; VIS
Summer	0.144	0.021	0.251	TEM; RHU; WIN
Autumn	0.209	0.044	0.495	PRS; RHU; WIN
Winter	0.473	0.224	0.124	TEM; PRS; RHU; WIN; VIS

Note: ** *p* < 0.01; * *p* < 0.05. The parameters were as follows: precipitation per hour (PRE, mm); average air temperatures (TEM, °C); average atmospheric pressure (PRS, hpa); average relative humidity (RHU, %); average wind speed of 10 min (WIN, m/s) and visibility (VIS, m).

**Table 3:** Comparison of fitted values and evaluation parameters for different ARIMA models.

(*p*, *d*, *q*)	Fitted Value	AIC	ME	RMSE	MAE	MPE	MAPE	MASE	ACF1
(*0*, *2*, *0)*	44,412.54	545.99	432.3713	12,361.73	8842.708	−8.91302	105.5697	1.428684	−0.53091
(*0*, *2*, *1)*	33,384.6	527.72	483.9527	7722.098	5739.791	−22.8288	60.53931	0.927357	−0.31949
**(*0*, *2*, *2)***	**26,238.82**	**523.42**	**720.3416**	**6537.497**	**4925.537**	**−29.9626**	**59.61555**	**0.795801**	**0.03333**
(*1*, *2*, *0)*	40,049.89	539.91	496.785	10,450.91	7624.494	−19.8323	89.65436	1.231862	−0.28397
(*2*, *2*, *0)*	27,482.05	533.29	252.3898	8654.897	6388.379	−27.5835	70.08172	1.032147	−0.14205
(*2*, *2*, *2)*	28,093.83	526.45	700.1022	6448.297	4708.604	−27.836	56.00602	0.760752	−0.057
(*2*, *2*, *1)*	26,988.48	525.95	472.1389	6666.584	5086.942	−27.9409	59.45506	0.821879	−0.07016
(*1*, *2*, *1)*	29,899.82	527.44	502.4846	7288.674	5579.637	−27.4644	63.05462	0.901482	−0.15394
(*1*, *2*, *2)*	28,553.56	524.83	740.2056	6524.263	4830.089	−28.8038	58.05218	0.78038	−0.04547

Note: The mathematical indicators were as follows: Akaike information criterion (AIC), mean error (ME), root mean squared error (RMSE), mean absolute error (MAE), maximum permissible error (MPE), mean absolute percentage error (MAPE, %), the mean absolute scaled error (MASE) and ACF1 (auto correlation of errors at lag 1). The bold format of the values represents the type of ARIMA selected in the study, due to its lower AIC, RMSE, etc.

**Table 4:** Comparison of evaluation parameters for different models.

(*p*, *d*, *q*)	AIC	ME	RMSE	MAE	MPE	MAPE	MASE	ACF1
(*0*, *1*, *0*)	1127.64	68.91637	8058.546	5822.154	−16.5185	50.52383	0.981823	−0.40433
(*1*, *1*, *1*)	1117.11	198.4567	7014.965	5393.174	−19.694	51.15323	0.909482	−0.01324
**(*0*, *1*, *1*)**	**1115.41**	**173.0956**	**7035.695**	**5323.927**	**−19.9934**	**50.98074**	**0.897804**	**0.031298**
(*1*, *1*, *0*)	1120.18	97.27034	7371.012	5222.038	−19.3682	49.95879	0.880622	−0.10025
(*0, 1, 2*)	1117.14	192.8843	7017.028	5385.995	−19.7073	51.08738	0.908271	−0.0086
(*2*, *1*, *0*)	1119.26	121.0036	7167.071	5176.703	−19.6148	49.82251	0.872977	−0.03197
(*2*, *1*, *1*)	1119.07	210.9474	7011.803	5392.778	−19.7556	51.31665	0.909415	−0.01143
(*2*, *1*, *2*)	1121.10	202.9406	7013.89	5395.582	−19.7244	51.23666	0.909888	−0.01233

Note: The mathematical indicators were as follows: Akaike information criterion (AIC), mean error (ME), root mean squared error (RMSE), mean absolute error (MAE), maximum permissible error (MPE), mean absolute percentage error (MAPE, %), the mean absolute scaled error (MASE) and ACF1 (auto correlation of errors at lag 1). The bold format of the values represents the type of ARIMA selected in the study, due to its lower AIC, RMSE, etc.

**Table 5:** Correlation analysis of weekly data of NAI concentrations with meteorological factors.

Period	Correlation and Significance	PRE_W	TEM_W	PRS_W	RHU_W	WIN_W	VIS_W
Week 1–Week 68	Pearson correlation	0.472 **	0.154	−0.384 **	0.471 **	−0.270	0.093
	Significance	0.000	0.209	0.001	0.000	0.026	0.452
	*n*	68	68	68	68	68	68

Note: ** *p* < 0.01. The PRE_W in this table is the hourly precipitation summed to a week, TEM_W stands for average weekly temperature, PRS_W represents the weekly average atmospheric pressure, RHU_W represents the weekly average relative humidity, WIN_W represents the weekly average wind speed, VIS_W represents weekly average visibility. TEM_W, PRS_W, RHU_W, WIN_W and VIS_W are calculated by averaging the raw hourly data over a week.

**Table 6:** Multiple linear regression analysis of meteorological factors and NAIs from 69 weeks.

Period	R	R^2^	Durbin-Watson	Meteorological Factors
Week 1–Week 68	0.591	0.349	1.574	PRE_W; PRS_W; RHU_W

## Data Availability

The data presented in this study are within the article.

## Supplementary Materials

The following are available online at https://www.mdpi.com/article/10.3390/ijerph18095037/s1, Table S1: Division of the week according to date and weekly average data of NAIs concentrations.
