# Supplementary material for: Different Time Scale Distribution of Negative Air Ions Concentrations in Mount Wuyi National Park
Source: Int J Environ Res Public Health. 2021 May 10;18(9):5037. doi: 10.3390/ijerph18095037 (PMC8126208; doi:10.3390/ijerph18095037)
Supplement: Supplementary file 1 [file ijerph-18-05037-s001.zip › ijerph-1137691-supplementary.pdf]

Table S1. Division of the week according to date and weekly average data of NAIs concentrations.

Week	Data	Week	Data	Week	Data	Week	Data
1	2018/10/01-2018/10/07	18	2019/01/28-2019/02/03	35	2019/05/27-2019/06/02	52	2019/09/23-2019/09/29
2	2018/10/08-2018/10/14	19	2019/02/04-2019/02/10	36	2019/06/03-2019/06/09	53	2019/09/30-2019/10/06
3	2018/10/15-2018/10/21	20	2019/02/11-2019/02/17	37	2019/06/10-2019/06/16	54	2019/10/07-2019/10/13
4	2018/10/22-2018/10/28	21	2019/02/18-2019/02/24	38	2019/06/17-2019/06/23	55	2019/10/14-2019/10/20
5	2018/10/29-2018/11/04	22	2019/02/25-2019/03/03	39	2019/06/24-2019/06/30	56	2019/10/21-2019/10/27
6	2018/11/05-2018/11/11	23	2019/03/04-2019/03/10	40	2019/07/01-2019/07/07	57	2019/10/28-2019/11/03
7	2018/11/12-2018/11/18	24	2019/03/11-2019/03/17	41	2019/07/08-2019/07/14	58	2019/11/04-2019/11/10
8	2018/11/19-2018/11/25	25	2019/03/18-2019/03/24	42	2019/07/15-2019/07/21	59	2019/11/11-2019/11/17
9	2018/11/26-2018/12/02	26	2019/03/25-2019/03/31	43	2019/07/22-2019/07/28	60	2019/11/18-2019/11/24
10	2018/12/03-2018/12/09	27	2019/04/01-2019/04/07	44	2019/07/29-2019/08/04	61	2019/11/25-2019/12/01
11	2018/12/10-2018/12/16	28	2019/04/08-2019/04/14	45	2019/08/05-2019/08/11	62	2019/12/02-2019/12/08
12	2018/12/17-2018/12/23	29	2019/04/15-2019/04/21	46	2019/08/12-2019/08/18	63	2019/12/09-2019/12/15
13	2018/12/24-2018/12/30	30	2019/04/22-2019/04/28	47	2019/08/19-2019/08/25	64	2019/12/16-2019/12/22
14	2018/12/31-2019/01/06	31	2019/04/29-2019/05/05	48	2019/08/26-2019/09/01	65	2019/12/23-2019/12/29
15	2019/01/07-2019/01/13	32	2019/05/06-2019/05/12	49	2019/09/02-2019/09/08	66	2019/12/30-2020/01/05
16	2019/01/14-2019/01/20	33	2019/05/13-2019/05/19	50	2019/09/09-2019/09/15	67	2020/01/06-2020/01/12
17	2019/01/21-2019/01/27	34	2019/05/20-2020/05/26	51	2019/09/16-2019/09/22	68	2020/01/13-2020/01/19
